# Exploring the Linkage Between Ruminal Microbial Communities on Postweaning and Finishing Diets and Their Relation to Residual Feed Intake in Beef Cattle

**DOI:** 10.3390/microorganisms12122437

**Published:** 2024-11-27

**Authors:** Pablo Peraza, Tamara Fernández-Calero, Hugo Naya, José Sotelo-Silveira, Elly A. Navajas

**Affiliations:** 1 Instituto Nacional de Investigación Agropecuaria, Las Brujas, Canelones 90100, Uruguay; pperaza@inia.org.uy; 2 Bioinformatics Unit, Institut Pasteur de Montevideo, Montevideo 11400, Uruguay; tamfer@pasteur.edu.uy (T.F.-C.); naya@pasteur.edu.uy (H.N.); 3 Departamento de Ciencias Naturales y Exactas, Universidad Católica del Uruguay, Montevideo 11600, Uruguay; 4 Departamento de Producción Animal y Pasturas, Facultad de Agronomía, Universidad de la República (UDELAR), Montevideo 12900, Uruguay; 5 Departamento de Genómica, Instituto de Investigaciones Biológicas Clemente Estable, Montevideo 11600, Uruguay; sotelojos@gmail.com

**Keywords:** Bos taurus, microbiome, feed efficiency

## Abstract

Feed efficiency significantly impacts the economics of beef production and is influenced by biological and environmental factors. The rumen microbiota plays a crucial role in efficiency, with studies increasingly focused on its relationship with different rearing systems. This study analyzed 324 rumen samples from bulls and steers categorized as high and low efficiency based on residual feed intake. The animals were fed two diets (postweaning and finishing) and rumen samples were sequenced using a reduced representation sequencing (RRS) based approach. The results indicated that diet significantly affected microbial diversity and abundance. In postweaning diets, Actinomycetota, particularly *Bifidobacterium*, were prevalent, aiding carbohydrate fermentation. In contrast, *Acetoanaerobium* was identified in finishing diets, likely contributing to acetate production. Additionally, *Bacteroides* and *Butyrivibrio* were abundant during postweaning, known for fiber degradation and volatile fatty acid production. Notably, *Prevotella* and *Fibrobacter succinogenes* were associated with high feed intake and nutrient utilization, indicating their potential as microbial biomarkers. However, alpha diversity indices showed no significant relationship with feed efficiency, suggesting that diversity alone may not adequately reflect the complexity of feed efficiency phenotypes. These findings highlight the importance of diet and microbial interactions on feed efficiency and suggest further research to explore these microbial contributions to precision feeding strategies.

## 1. Introduction

Feed efficiency is relevant for the beef cattle industry with a significant impact on economic and environmental sustainability. Residual feed intake (RFI) is one of the most investigated traits to assess feed efficiency, which is defined as the difference between the observed and the expected feed intake based on animal performance [1]. It stands out as a valuable trait for improving livestock feed efficiency due to its low to moderate heritability and independence or low phenotypic and genetic associations with production traits [2,3]. Additionally, the investigation of the physiological mechanisms underlying variations in feed efficiency has also focused on RFI [4,5,6]. Research studies show that feed efficiency is a multifactorial trait explained by different biological and environmental factors [2], and the composition of the rumen microbiome is increasingly recognized as a critical factor potentially influencing this trait [7,8]. Recent studies have highlighted the significant relationship between rumen microbiome structure and the ability of ruminants to convert feed into energy efficiently [9,10,11]. As the primary site for feed fermentation in cattle, the rumen harbors a diverse microbial population that plays a substantial role in nutrient metabolism and energy extraction [5]. It has been suggested that efficient animals have a less diverse microbiome, producing large amounts of fermentation by-products for animal energy production [12]. Various microorganisms such as Clostridiales, *Prevotella*, *Ruminococcus*, *Lachnospiraceae*, *Fibrobacter*, *Anaerovibrio*, and methanogens, among others, have been described as correlated with high- or low-efficiency characteristics, although many of these have not always been verified across studies [10,13,14]. This indicates the great versatility of different microorganisms and how the difference may lie at even lower taxonomic classifications (below species).

Studies of rumen content have shown that microbiome communities are strongly affected by dietary differences, especially in association with fiber/concentrate ratios [15,16]. Beef cattle finishing diets typically include high levels of concentration, with a strong effect on rumen metabolism, including the archaea populations responsible for capturing fermentation products for methane production [17,18]. In a study by [19], cattle fed with different concentrate–forage ratios in the diet showed higher community diversity indices in animals fed the lower concentrate diet, with a significant influence of diet-specific changes in taxa abundance, particularly Bacillota, Bacteroidota, and Pseudomonadota phylum, and large differences in the protozoan and fungal composition were highlighted. This was also observed by other studies, where an increase in the proportion of grain and starch in the diet leads to a lower bacterial richness and diversity in the rumen, possibly due to a reduction in the ruminal pH [20,21]. Consequently, shifts in microbial diversity may have implications for rumen function with an impact on the overall cattle health and feed efficiency.

Differences in the rumen metagenomic profiles in cattle with high and low feed efficiency suggest that it could be possible to identify potential microbial markers linked to feed efficiency. Several sequencing strategies have been used to study the rumen microbiota, providing a list of candidate taxa at various taxonomic ranks and suggesting possible mechanisms explaining efficiency differences, while fewer studies report a list of candidate microbial genes [5,22]. The restriction enzyme–reduced representation sequencing (ER-RRS) methodology before sequencing is a low-cost method recently proposed by [23].

This technique, commonly referred to as genotyping-by-sequencing (GBS), involves extracting and isolating genetic material (DNA) from a sample, fragmenting it with one or more restriction enzymes, and selecting fragments within a specific size range. The selected fragments are then amplified and sequenced using high-throughput sequencing platforms. Other authors have already used this method [24,25] and analyzed the characteristics of the rumen microbiota, as opposed to the classical amplicon amplification or shotgun sequencing methods.

The present study explored the compositional profile of rumen content in Hereford cattle using the ER-RRS methodology with the aim of investigating the impact of two contrasting diets (postweaning vs. finishing), the effect of gender (bulls vs. steers), and the differences between high- and low-efficiency animals. In this study, we present results showing the impact of dietary influences on cattle microbial diversity, with higher diversity when animals were fed with finishing diets. A moderate shift in the overall differences between high- and low-feed efficiency animals could also contribute to identifying potential rumen markers or taxa as proxies for this trait. In our results, we did not observe lower microbial diversity in more efficient animals compared to less efficient animals.

## 2. Materials and Methods

### 2.1. Animal Management, Feeding Diets, and Residual Feed Intake Tests

The animal management and experimental procedures, as well as the collection of rumen content for this study, were approved by the INIA Honorary Committee on Ethics in the Use of Animals (resolution INIA2018.11).

Data used in this study were recorded in 2021 and 2022 in 257 purebred Hereford steers and 57 bulls at the Hereford Central Test Station in Kiyú, San José, Uruguay (34.6° S, 56.7° W), where feed efficiency tests were carried out as part of a large national project on the genetic improvement of feed efficiency in the Hereford breed [26].

Postweaning RFI was evaluated in a cohort of bulls (BPW) and two cohorts of steers (SPW1; SPW2), while finishing RFI was assessed only in the same cohorts of steers (SF1; SF2) after postweaning tests. Table 1 describes the number of animals in each group, the composition, and the analysis of diets used.

After 28 days of acclimatization to the diet and feeding system, RFI tests were conducted. Individual feed intake was recorded in 70-day tests using an automated feeding system (Vytelle, GrowSafe Systems Ltd., Calgary, AB, Canada) with ad libitum access to feed and water [3]. Dry matter intake (DMI) was calculated by adjusting for the weekly dry matter (DM) analysis of the diet. The animals were weighed fortnightly to calculate their average daily gain (ADG), and certified technicians measured subcutaneous backfat thickness (BFAT) at the end of the RFI test. Phenotypes for RFI were estimated as the difference (or residual) between the actual feed intake and the expected feed intake using the following linear regression model:

DMI = b_0_ + b_1_MBW + b_2_ADG + b_3_BFAT + Test + e, (1)

where DMI is the dry matter intake (kg DM/day), MBW is the metabolic body weight (BW 0.75), ADG is the average daily gain obtained by linear regression of weights recorded fortnightly during the test (kg/day), BFAT is ultrasound back fat at the end of the test (mm), b_0_ is the intercept, and b_1_, b_2_, and b_3_ are the partial regression coefficients of each trait on DMI and the residual (e) represents the RFI. Based on the mean and standard deviation (SD) of RFI, the bulls (BPW), steers in the postweaning (SPW), and steers at finishing (SF) were classified into two groups: high-efficiency (Low RFI—LRFI) and low-efficiency (High RFI—HRFI) animals. In all cases, LRFI and HRFI were those with RFI values 0.5 SD below and above the mean, respectively. The performance of LRFI and HRFI groups was characterized by the analysis of variance of RFI, DMI, ADG, MBW, and BFAT, and means were compared by Tukey’s HSD (Honestly Significant Difference).

### 2.2. Ruminal Content Sampling and Sequencing

We collected ruminal samples within two days after the end of each RFI test. We took two mornings (while the animals were still on the same diet), during which the animals were usually removed from the pen at 6 a.m. We started the process at 8 a.m. to avoid receiving diluted ruminal samples. Raw ruminal fluid was collected by sampling through an oral tube with dimensions of 18 mm in diameter and 220 mm in length, connected to a manual pump. To avoid cross-contamination, individual tubes were used for each animal, and the pump was thoroughly cleaned between animals. Samples of the solid and liquid fractions of the rumen content were collected in two 50 mL tubes, where pH and weight were measured. To preserve the integrity of the rumen sample after collection, the tubes were immediately placed on dry ice to ensure complete freezing. The samples were transported to the laboratory and stored at −80 °C until further processing.

### 2.3. DNA Extraction, Fragment Sequencing and Bioinformatic Analysis

Whole rumen contents were lyophilized using a Labconco Freeze Dry System/Freezone^®^ 4.5 instrument (LABCONCO Corporation, Kansas City, MO, USA) at the Animal Genomic DNA Bank at INIA and then shipped to AgResearch in Mosgiel, New Zealand for DNA extraction and the subsequent sequencing. Microbial samples were processed using the methodology described in [23]. Briefly, DNA was extracted using a combined bead-beating, phenol, and column purification protocol and sequenced using restriction enzyme reduced representation sequencing (RE-RRS) using the restriction enzyme PstI (CTGCA|G). Sequencing was performed on an Illumina NovaSeq 6000, (Illumina, Inc., San Diego, CA, USA) which was used to generate single-end reads of 91 bp length. Raw sequences were processed using Bowtie2 v1.0.0 to remove Bos taurus sequences as contaminants in all the samples [27,28] and then a quality control filtering was applied using Trimmomatic v0.38 [29] to remove low quality and reads < 40 pb long for further processing. Both analyses were performed using the OmicsBox v3.0.30 software (OmicsBox—Bioinformatics Made Easy, BioBam Bioinformatics, https://www.biobam.com/omicsbox, accessed on 3 March 2019). The sample reads were processed using Dada2 v1.21 software [30] to obtain a list amplicon sequence variant (ASV), and then we retained only those ASV present in more than 50% within each cohort/group. After several tests (10%, 25%, and 50%), this option was chosen to generate a microbiota matrix with a computationally manageable number of ASVs and a reduced number of zeros on each taxon. The resulting ASV sequences were mapped on Kraken2 [31,32] by default parameters against two complementary databases: the Kraken Standard PlusPF https://benlangmead.github.io/aws-indexes/k2, accessed on 1 September 2024) (Refeq archaea, bacteria, viral, plasmid, protozoa, and fungi indices) and Cow Rumen v1.1. (EMBL-EBI) databases [33]. Complete taxonomic information on these ASVs was obtained from an extension of the NCBI Taxonomy using Taxallnomy (http://bioinfo.icb.ufmg.br/taxallnomy/, accessed on 1 September 2024) [34]. To generate the taxonomy matrix, for each mapped ASV, we filled the empty fields in phylum, Class, Order, family, genus, or species with the immediately preceding known field of taxonomy. For example, if an ASV is classified as Bacteria at the superkingdom level, but the subsequent levels are empty, it will appear as Phy_in_Bacteria, Fam_in_Bacteria, and Gen_in_Bacteria at the phylum, family, and genus levels, respectively. Different R v4.2.0 packages were used for microbiome data interpretation, such as phyloseq [35] for bar plots, taxa proportion calculations, alpha and beta analysis and clustering, and Microbiota Process v1.16.1 [36] and ANCOM-BC2 v2.8.0 [37] for differential analysis. For the differential analysis of taxa between diets, we only used data from the steers (on postweaning and finishing) for both years, and the model used in ANCOMBCII was y = Diet + year, where diet included postweaning and finishing, and the two years (2021 and 2022) were considered. A differential analysis between the RFI groups was performed separately on the bulls and then on the steers in each of the diets, where, in this case, we also used the year variable: y = RFI group + year. ANCOM-BC2 performs a bias correction by a compositional log transformation of the data without a normalized input, and we plotted the results from analyzed taxa using the option “bias_correct_log_table” to obtain a normalized data count. The results were considered significantly different when the *p* value < 0.05. The most influential microbial genera from each analysis (diet, postweaning vs. finishing) or (RFI group, HRFI vs. LRFI) were selected for discussion. We then performed an ordination analysis of the data using nMDS as ordination types with the Bray–Curtis index as a dissimilarity measure and a redundancy/PERMANOVA analysis by using addRDA function in “mia” package v1.13.47, https://github.com/microbiome/mia, accessed on 1 September 2024) using a relative abundance transformation for the calculation of explained variances.

## 3. Results

### 3.1. Feed Efficiency Data for Each Trial

To characterize the rumen microbial composition of Hereford cattle and explore potential differences between postweaning and finishing diets, as well as between high and low feed efficiency, we first selected three groups of animals: bulls (BPW) and steers on postweaning diets (SPW) and steers on finishing diets (SF). The steers were analyzed in two different years, adding another variable to the experiment. While only the SPW and SF groups were used to explore the differences in rumen composition between the diets, all the groups were used to analyze the differences between the high and low feed efficiencies. No significant differences in chemical composition were observed between the diets from each rearing system (Table 1).

The descriptive statistics of DMI, ADG, MBW, BFAT, and RFI are presented in Table 2 for all five groups. The comparison of the animal performance of LRFI and HRFI for BPW, SPW, and SF (Table 3) shows that in all cases, DMI was significantly lower in the LRFI animals compared to the HRFI group, while non-significant differences were found for the other traits except for BFAT in SPW. When comparing the LRFI and HRFI animals, the efficient animals have an average intake of about 10% lower than the less efficient animals. This approach enabled us to accurately define differential sub-groups for feed efficiency.

### 3.2. Sequencing Results and Taxonomic Assignment

**Figure 1:**
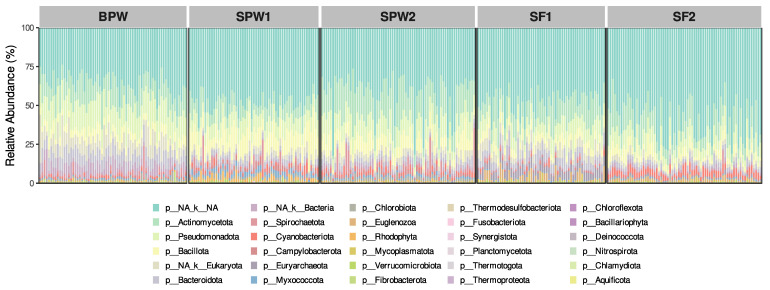
Bar chart composition for phylum representation from different groups. Bar chart composition at the phylum level for the five groups analyzed. Each bar represents a sample and the different colors a different phylum. BPW: bulls in postweaning; SPW: steers in postweaning; SF: steers in finishing. The numbers refer to the year of the test.

ER-RRS was performed on the DNA extracted from the ruminal samples to explore the ruminal microbial composition. An average of 384 k, 422 k, 579 k, 789 k, and 581 k sequences were obtained for each group, BPW, SPW1, SPW2, SF1, and SF2, respectively. The raw reads were quality-filtered, and host genome (Bos Taurus) reads were removed. Table S1 resumes the remaining sequences used for the subsequent analysis. We next identified unique amplicon sequence variants (ASVs) and retained those present in at least 50% of the samples within each group. This resulted in a sequence loss of 35–41% in each group, identifying a total of 28,651 ASVs in the 327 samples. These ASVs were mapped for taxonomic identification combining the RefSeq and CowRumen 1.1 databases. Two ASVs were considered artifacts generated by the ER-RRS strategy and removed from the dataset. This decision was based on the fact that (i) these sequences comprised 45–70% of the reads in most samples, (ii) they were not identified in our selected databases, and (iii) we did not find precedents in recent analyses [38,39] describing microorganisms dominating ruminal microbial communities in such abundance. Tables S3 and S4 show ASV superkingdom domain classification into bacteria, eukaryotes, archaea, and other entries (plasmids and transposons). As expected, most ASVs were mapped to bacteria, followed by eukaryotes, with archaea being the minority group. It is interesting to note that eukaryotes may be overrepresented, as these genomes tend to be larger compared to prokaryotes, possibly making restriction enzyme digestion more likely [40]. In Figure 1 we present a bar plot of the composition at the phylum level of each group.

The taxonomic analysis showed that within the identified ASVs, the most abundant phyla across groups were Actinomycetota (Actinobacteria), Bacillota (Firmicutes), Bacteroidota (Bacteroidetes), Pseudomonadota (Proteobacteria), Campylobacterota, Cryptophyceae, Cyanobacteriota, Chlorobiota, and Spirochaetota (Table 4).

At the genus level, 426 different taxa were identified, with *Azorhizobium*, *Prevotella*, Chooromonas, *Streptococcus*, and *Mycobacterium* among the most abundant in almost all the samples (Table 5). *Azorhizobium caulinodans* were the most abundant species in all the groups. Among prokaryotes, the Archaea domain is of particular interest in the rumen since it comprises methanogenic microorganisms relevant to methane emissions. The only methanogenic Archaea reported at over 0.5% at the genus level was *Methanocorpusculum* detected only in the SF1 group. At a deeper level, *Methanogenium cariaci* and *Metopus palaeformis endosymbiont* were identified at the species level.

### 3.3. Diversity Analysis

**Figure 2:**
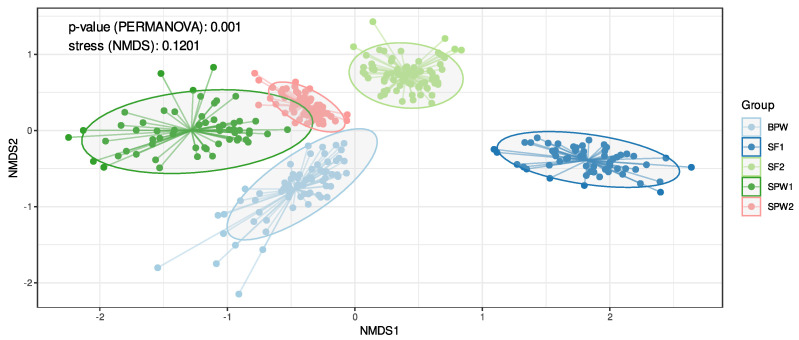
Structure analysis for the composition from the different groups. NDMS plot of all the samples analyzed in the study. In light blue are the bulls (BPW) and in dark green and pink there are the steers, all on the postweaning diet for years 1 and 2 (SPW1 and SPW2). In dark blue and light green are the steers on the finishing diet, also for years 1 and 2, respectively, (SF1 and SF2).

To better understand microbial community similarities between groups, a non-metric multidimensional (NMDS) analysis was performed (Figure 2). NMDS ordination was in two dimensions with a stress value of 0.1204, indicating ordination is reliable. The ordination in the first dimension relates to the dietary groups where the postweaning diet groups (BPW, SPW1, and SPW2) are separated from the two finishing groups (SF1 and SF2). The second dimension relates to the year the experiment was performed (BPW, SPW1, SF1/SPW2, and SF2) (Figure 2). A PERMANOVA analysis was performed to test the importance of each variable on the similarity between samples. Diet, year, and category explain 13.4%, 25.6%, and 2.3% of the variance, respectively, and all the factors showed statistical significance (*p* value = 0.001).

**Figure 3:**
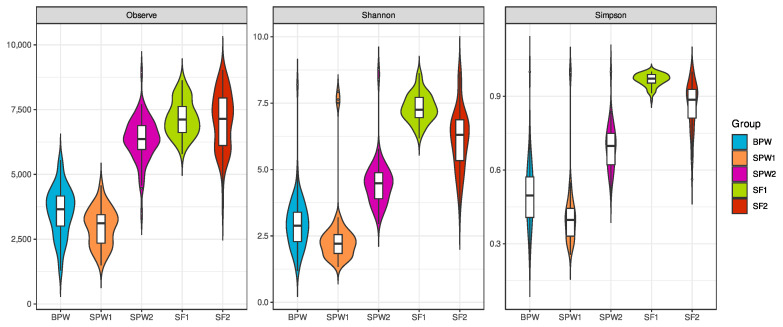
Different alpha diversity indices for all the rumen sample groups. Richness, Shannon, and Simpson diversity indices from all groups of samples. Colors represent a group of samples. BPW: bulls in postweaning; SPW: steers in postweaning; SF: steers in finishing.

Alpha diversity analyses using Shannon’s (accounts for both the abundance and evenness of species in a community) and Simpson’s (1—the probability of observing two bacteria in a community by chance while belonging to different species) indices showed that higher community diversity is seen with the finishing diets (SF1 and SF2) than with the postweaning diets (BPW, SPW1, and SPW2) as shown in Figure 3. Overall, the Shannon index values averaged 6.73 for the finishing and 3.37 for the postweaning diets, with values above 3 usually indicating high community diversity [41]. Simpson’s index also showed a greater diversity dispersion in the finishing diets. The alpha diversity indices are also impacted by the year when the samples were taken, with the samples from the second year being significantly more diverse than the ones from the first one. Nevertheless, although attenuated, differences in the alpha diversity indices between the diets within the second year remain significant. Altogether, these results confirm that both diet and year have an impact on the rumen microbial community diversity and composition.

Next, we investigated the diversity between HRFI and LRFI. Since HRFI and LRFI groups were defined within each experimental group (BWP, SWP1, SWP2, SF1, and SF2) and the PERMANOVA analysis results suggest that the factors defining each group (diet, category, and year) are shaping the microbial composition, we compared the alpha diversity indices between the HRFI and LRFI animals within each experimental group. The bulls and steers from the first year showed significant differences in the alpha diversity indices between the HRFI and LRFI animals (Table 6).

The bulls only had significant differences in observed richness and the steers in all the diversity indices. Other groups had minor differences in observed richness without significance. The Shannon and Simpson indices were significantly lower in the LRFI animals. Overall, the differences in alpha diversity between the HRFI and LRFI are not consistent and do not appear to depend solely on diet type or environmental conditions. Instead, a combination of factors may ultimately determine whether the diversity indices differ between the groups.

### 3.4. Differences in Microbiota Abundances

To understand if changes in the abundance of taxa could be associated with diet, we performed a differential abundance analysis using ANCOMBC v2.8.0. Only steers were considered for this analysis. The complete list of species identified as differentially abundant in each diet is available in Table 7.

**Figure 4:**
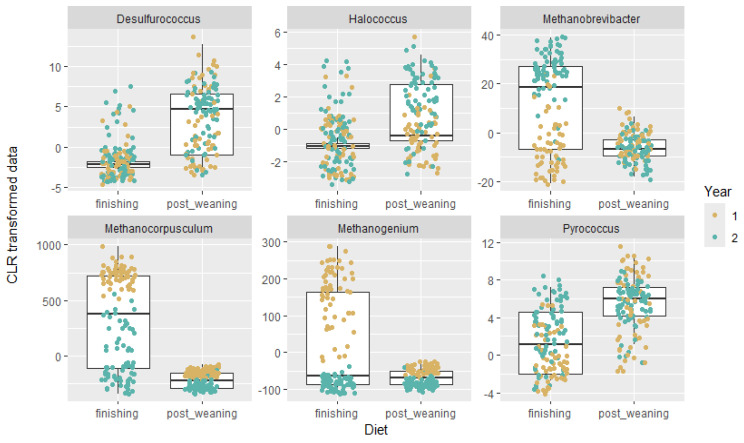
Archaea distribution between the postweaning and finishing diets. A box plot analysis of the six Archaea present in the samples in both diets. Colors represent the year of the RFI test (brown = Year 1 and green = Year 2).

Acetoanaerobium sticklandii, Metopus palaeformis endosymbiont, Stella humosa, Flexibacter flexilis, and Nocardia globerula are among the species overrepresented in the animals on the finishing diet—meanwhile, Clostridioides mangenotii, Myxococcus fulvus, Streptococcus anginosus, Prevotella sp. Rep29, Spe_in_Chlorobiota, and Azospirillum brasilense were found to be significantly overrepresented in the animals on the postweaning diet. Again, we focused on the Archaea group and analyzed all the genera that were detected as differentially abundant between the postweaning and finishing diets (Figure 4).

Desulfurococcus, *Halococcus*, and Pyrococcus were found to be significantly overrepresented in the postweaning diet, meanwhile *Methanobrevibacter*, *Methanocorpusculum*, and *Methanogenium* in the finishing diet. Although these genera belong to the Archaea domain, *Methanocorpusculum*, *Methanobrevibacter*, and *Methanogenium* belong specifically to methanogens. These are microorganisms that produce methane as a by-product of their metabolism, typically under anaerobic (oxygen-free) conditions. The remaining archaea (*Halococcus*, *Pyrococcus*, and *Desulfurococcus*) are further classified into other categories as hyper thermophilic and halophilic archaea. When comparing diets, in some of these cases, we observed an increase only in one of the years, without consistent patterns across both years. This variation may reflect the adaptability of the rumen, suggesting that other factors -such as environmental conditions, climate, or animal genetics- could influence the presence and abundance of certain rumen species.

Although no significant differences were found in the alpha diversity indices between the LRFI and HRFI groups, we explored if specific taxa could be differentially associated with them. For the differential taxa abundance analysis between the LRFI and HRFI animals, we selected three different groups. For different diets and categories, we independently analyzed the postweaning diet bulls (BPW) and steers (SPW: from both years) and the finishing diet steers (SF: from both years). Table 8 and Table 9 show the differentially abundant taxa identified within each group.

## 4. Discussion

In the present work, we analyze the composition and differential abundance of feed efficiency groups of cattle using the ER-RRS methodology. Although taxonomic identification from sequences generated by RRS in metagenomic studies, as described by [23], is constrained by both the technique and the limited completeness of reference databases, our approach to identifying ASVs—as tags were referenced in the article—for metagenomic profiling has been carefully designed to balance processing efficiency and the retrieval of accurate information by leveraging reliable databases.

The group of [42] explored the potential of double digestion to simplify metagenomic datasets, finding that the estimated relative abundances achieved were comparable to those obtained through shotgun metagenomics. They found that the bias introduced by reduced representation was lower than the bias resulting from DNA extraction or GC content (guanine (G) or cytosine (C) content). As an amplicon sequencing technique, relative abundances estimated for different taxa have been reported to correlate well with other methods, such as 16S rRNA sequencing, but showing some discrepancies in terms of presence and absence, maybe due to a rarefaction transformation [24]. Still, care must be taken in the analysis and discussion of each taxon due to the nature of the sequencing method.

### 4.1. Effects of Diversity on Microbiota Abundances

There is evidence that changes in diet composition can lead to variations in taxon abundance in cattle [15,43]. High-grain diets in dairy and beef cattle have been associated with declines in bacterial diversity in the rumen due to a pH reduction derived from diet composition, where species struggle to survive in an acidic environment [44,45]. In fact, lower pH has been found in the rumen samples of animals fed finishing diets (with a higher grain content) than in those fed postweaning diets, mainly due to carbohydrate fermentation in the rumen, mostly producing organic acids such as lactate and volatile fatty acids (VFAs) [20,46] leading to this pH reduction. In our study, as expected, the pH values were lower in the animals fed with finishing diets than in those under postweaning diets (Table S2). However, diversity indices and analyses show higher microbial diversity in the finishing diets (Figure 3), in opposition to what was reported by [45,47]. Nevertheless, ref. [44] showed that some diversity indices did not consistently correlate with a decrease in pH and bacterial abundance, suggesting that the rumen may adapt to long-term feeding with high-concentrate diets to reduce unfavorable effects on bacterial diversity. The diversity reduction in postweaning compared to finishing diet found in our study was consistent for both years.

In this study, a higher proportion of the genus Bacillota was found in postweaning diets, as observed in previous research [19]. Among these is the genus *Butyrivibrio*, which, in addition to butyrate production, is important for fiber degradation, protein degradation, lipid biohydrogenation, and the production of microbial inhibitors [48].

We also found that *Bifidobacterium* was abundant in the postweaning animals. The Actinomycetota phylum is crucial in cattle’s rumen microbial population dynamics during adaptation to high-grain diets. The relative abundance of Actinomycetota in the rumen of dairy cattle, for example, is significantly increased during subacute ruminal acidosis, highlighting the influence of diet on the rumen microbiota [49]. In our study, *Bifidobacterium* was one of the Actinomycetotas present in the postweaning diet. This organism possesses a diverse set of glycosyl hydrolases, allowing access to different dietary glycans and potentially facilitating the fermentation of multiple carbohydrates [50]. From the Pseudomonadota list, the genus *Aeromona* appears to be differentially abundant in postweaning, not being a major component of the rumen community, but found in other studies and identified as a pathogen [51]. Other genera within Pseudomonadota include *Klebsiella*, *Methylobacterium*, *Pseudomonas*, and *Succinivibrio*. *Succinivibrio* plays a significant role in the fermentation process (carbohydrate fermentation, Nitrogen Utilization, and Supporting Rumen Health). These bacteria are known for their ability to ferment carbohydrates, producing important fermentation end-products like succinic acid and acetic acid [52].

Within the Bacteroidota, which was significantly abundant in the postweaning stage, *Bacteroides* play a key role in digestion. These species are adept at breaking down complex carbohydrates, essential for fermentation, and aid in the degradation of plant materials, producing key short-chain fatty acids like acetate, propionate, and butyrate [53].

*Euglenozoa* was significantly higher in the finishing diets at the genus level than in postweaning. These organisms have previously been reported in rumen metagenomic studies in Holstein cattle, where maize is the main component; *Euglenozoa* appears in higher abundance in animals with low milk protein yield [54]. This might suggest that the genus *Euglenozoa* would prefer a diet with high levels of starch and carbohydrates to be developed in the rumen. These phyla play many roles, but how they function in the rumen still needs to be discovered.

*Acetoanaerobium*, a genus within the Bacillota phylum, appeared in the finishing diet. Although its role in the rumen has not been well documented, it may play a significant part in fermenting H₂ and CO₂ to acetate [55], indicating that its metabolism could contribute to acetate formation in high-concentrate diets. Besides producing acetate, *Acetoanaerobium* species may contribute to the balance of the rumen microbiome by interacting with other microorganisms, supporting efficient feed breakdown, and potentially influencing the production of gases like methane [56].

The presence of cyanobacteria has been reported in the rumen of cattle in several studies [5,57,58]. What we found in all the groups, but in low abundance, were sequences corresponding to the identification of *Microcystis*, *Anabaena*, *Chlorogloeopsis*, *Microchaete*, *Phormidium*, *Spirulina*, *Synechocystis*, *Geminocystis*, *Tolypothrix*, and *Trichormus* genera and the families Nostocales and Pseudanabaenaceae. Cyanobacteria may be introduced by ingesting eutrophic water or legumes, although their role in the cattle rumen microbiome and its interactions warrants further investigation.

Thus, the main metabolic difference between the diets is the emphasis on diverse carbohydrate fermentation pathways in the postweaning versus increased acetate production in the finishing diets. This shift in microbial populations reflects the rumen’s adaptation to maximize energy extraction based on diet composition.

The analysis of the steers across years revealed variable patterns of microbial diversity and taxonomic abundance, reflecting a lack of consistency in the relationship between microbial composition and feed efficiency over time. Significant differences in diversity indices between efficiency groups occurred only in the postweaning diets of the steers in one year, with no similar trends observed in the following year. This inconsistency suggests that the variable year may influence the rumen microbiota from year to year, affecting the relationship between microbial diversity and feed efficiency.

### 4.2. Microbiota Association with Feed Efficiency

Certain studies have shown a relationship between the alpha diversity index of the rumen microbiota in cattle and their feed efficiency [12]. However, the variability in the alpha diversity indices in our study did not indicate a significant association with the RFI in the animals (Table 6). Other studies observed that alpha diversity indices did not differ between high- and low-efficiency animals in dairy and beef cattle, suggesting that maybe these diversity indices may not be a significant parameter to differentiate feed efficiency phenotypes [13,59].

Since we had only one group of bulls, their data was processed separately from that of the steers (Table 8). At the genus level, the differential analysis revealed that in the HRFI group of the bulls, the most abundant taxa were *Actinomyces*, *Lactococcus*, *Leptospirillum*, *Mammaliicoccus*, and *Legionella*, among others. *Actinomyces* were identified in the rumen of cattle and reported to be a ureolytic bacterium in the rumen [60], and other studies have found that *Actinomyces ruminicola* sp. from the rumen of cattle were unable to hydrolyze urea [61] but hydrolyzed xylan and starch and fermented different types of sugars [62]. Additionally, *Actinomyces succiniciruminis* and *Actinomyces glycerinitolerans* have been associated with cellulolytic and xylanolytic activities [63]. While some species of *Lactococcus* are considered transient bacteria introduced with the feed, others, like *Lactobacillus ruminis* and *Streptococcus equinus*, are regarded as actual rumen inhabitants [64]. Research has explored the potential of *Lactococcus lactis* in reducing methane emissions in dairy cows, indicating its role in mitigating environmental impacts [65]. Moreover, *Lactococcus lactis* has also been investigated for its ability to scavenge oxygen in specific pathway metabolisms, which could benefit certain applications [66]. A potential area for future research is the metabolic activity of *Lactococcus* in the ruminal environment. *Lactococcus* species are known for their ability to ferment carbohydrates and produce lactic acid, which affects the pH balance in the rumen and plays a relevant role in nutrient absorption. Understanding how *Lactococcus* fermentation processes interact with the host’s digestive physiology could provide valuable insights into how these bacteria contribute to feed efficiency. Also, the plasmid pAC27 was reported as differentially overrepresented in this group. This plasmid is a genetic element that carries genes responsible for the degradation of chlorocatechols, facilitating the breakdown of chlorinated aromatic pollutants in bacteria such as 3-chlorobenzoate and chlorocatechols [67]. So far, there are no reports of these findings in cattle rumen. In the LRFI group, *Ruminococcus*, *Prorocentrum Legionella*, and two plasmids (Plasmid pVA380-1 and PlasmidRSF1010) were overrepresented by taxa.

In the HRFI group of steers in postweaning diets, *Mycolicibacterium diernhoferi*, *Thermochromatium tepidum*, and *Acetobacter pasteurianus* were detected, and in the finishing stage, the genus *Achromobacter* was identified. Acetobacter is a genus of acetic acid bacteria that plays a role in creating an anaerobic environment in the rumen, facilitating the growth of anaerobic bacteria and archaea, which are essential for efficient fermentation processes [68]. Additionally, studies have highlighted the presence of *Acetobacter* in the rumen microbiome, where its abundance may impact feed efficiency in beef cattle [69]. The greater relative abundance of *Acetobacter* in cows has been noted for its potential significance in rumen function, producing ruminal acetate, allowing the animal to obtain energy for productive purposes and, by extension, feed efficiency [70]. Other studies have detected *Acetobacter pasteurianus* in the rumen of dairy cows with laminitis, a painful systemic disease with significant welfare implications. This condition not only affects tissues but also damages the animal’s general condition [71].

No studies reported have previously shown the presence and abundance of *Mycolicibacterium diernhoferi* in the rumen microbial community of cattle. *Mycolicibacterium* genera report having arylsulfatase, nitrate reductase, and iron uptake activities. Most species are saprophytic (process decaying organic matter for nutrients), and some have been reported to cause infections and diseases. There are studies where microbial genes related to pathogenic activities were detected in low-efficiency animals [69]. *Achromobacter* was reported in the HRFI group in the finishing stage. Members of the Alcaligenaceae family have been identified in a variety of habitats, from soil to animals, where they can cause clinically relevant infections in animals. This microorganism from the Pseudomonadota phylum, declared as a human opportunistic pathogen, was detected in the gut of children with autism and gastrointestinal dysfunction [72].

Several microorganisms have been identified in the highly efficient animals between the bulls and steers, such as *Brevibacterium epidermidis*, *Bifidobacterium longum*, *Prevotella* communis, *Fibrobacter succinogenes*, *Streptomyces roseoverticillatus*, and others (Table 9). The *Prevotella* genus appears to be one of the most abundant taxa in the rumen. It contains numerous metabolically diverse bacterial species capable of growing on starch, proteins, peptides, hemicellulose, and pectin [73]. The occurrence in the LRFI group supports previous reports on its positive association with RFI in beef and dairy cattle [10,74]. The proportion of *Prevotella* in the gut microbiota has been positively correlated with traits such as feed intake, feed efficiency, and body weight gain and could potentially be used as a microbial biomarker [75]. *Fibrobacter succinogenes* is a predominant cellulolytic bacterium with high cellulolytic activities in the rumen, where its relative abundance has been linked to variations in feed components such as neutral detergent fiber and starch [76]. Bifidobacterium longum, a probiotic bacterium, has been studied for its potential role in the rumen environment. In addition, *Bifidobacterium longum* has been recognized for its probiotic properties, such as boosting immunity, preventing infection, and treating inflammatory diseases [77]. A study by [78] showed that the oral administration of *Bifidobacterium* improved both daily weight gain and feed conversion ratio in young calves. More recently, *Bifidobacterium* was found to be significantly more abundant in the rumen of efficient lambs when fed with a concentrate-based diet [79]. Our results are consistent with these studies and suggest that Bifidobacterium may be important in maximizing energy extraction from the diet.

Streptomyces, a genus of bacteria known for its ability to produce various bioactive compounds, has been linked to cattle feed efficiency through ionophores. Our study identified *Streptomyces roseoverticillatus* as abundant in the LRFI group. Monensin, another ionophore antibiotic from *Streptomyces*, is a widely used feed efficiency enhancer commonly used in confined cattle [80]. *Streptomyces* have been studied for their potential role in improving feed efficiency in livestock, where some strains can produce enzymes that break down cellulose and hemicellulose into simpler forms that are easily digestible by animals. *Streptomyces* can improve the overall efficiency of nutrient utilization in an animal’s digestive tract, resulting in better feed efficiency and potentially improved growth performance and producing metabolites with antimicrobial properties that help maintain a healthy gut microbiota to support optimal feed efficiency. However, it has not been reported in previous metagenomic analyses related to feed efficiency.

This study highlights significant microbial and metabolic differences between high and low feed efficiency groups (HRFI and LRFI) in cattle. The HRFI animals had an increased abundance of certain taxa, such as *Prevotella* and *Fibrobacter succinogenes*, which are known to efficiently break down fiber and carbohydrates to produce volatile fatty acids (VFAs) that support energy utilization. In contrast, the LRFI animals harbored a greater presence of taxa involved in less efficient metabolic pathways and, in some cases, potential opportunistic pathogens that may contribute to energy diversion towards immune responses rather than productive metabolism. These differences highlight the critical role of the rumen microbiome in influencing feed efficiency and suggest potential microbial biomarkers that could provide information on nutrient absorption and energy efficiency in beef production.

## 5. Conclusions

This study has provided valuable insights into the effects of diets on the rumen of beef cattle and their relationship to feed efficiency. In terms of changes in population structure, as measured by the diversity of the populations in the diets used, we observed an increase in diversity in our finishing diets. We identified the genera *Butyrivibrio* (Bacillota), *Bacteroides* (Bacteroidetes) and *Methylobacterium*, *Pseudomonas*, and *Succinivibrio* (Pseudomonadota) in the postweaning diets, suggesting an improved and controlled anaerobic environment for ruminal fermentation. In the finishing diets, we characterized *Acetobacter* (Proteobacteria) and Acetoanaerobium (Bacillota) influencing the balance of the rumen microbiome and interacting with other microorganisms to maintain a stable fermentation environment.

In terms of differences in feed efficiency, several genera have been described for the high-efficiency groups, such as *Streptomyces*, *Prevotella*, *Fibrobacter*, and *Bifidobacterium*, which improve the ability of feed to be converted into energy and assimilated by the animal. On the other hand, *Mycolicibacterium*, *Acetobacter*, and *Achromobacter* have been identified as predominant in low-efficiency animals, where only Acetobacter has a clear role with a negative impact on feed efficiency. These differences in the ruminal microbiota may contribute to the host feed efficiency, although this effect may be modulated to some extent by the type of diet offered. This study presents new data on the impact of dietary influence on microbial diversity in beef cattle and evidence of the overall differences between high and low-feed efficiency animals to identify potential taxa as proxies for this trait to be used as potential markers in the industry, where further research is needed for a comprehensive understanding of rumen dynamics.

## Figures and Tables

**Table 1:** Diet composition and chemical analysis in postweaning and fattening diets.

Group	BPW	SPW1	SPW2	SF1	SF2
Category	Bull	Steer	Steer	Steer	Steer
Animals	67	59	70	58	70
Year	1	1	2	1	2
Diet Composition (%)	Postweaning	Postweaning	Postweaning	Finishing	Finishing
Corn Silage	78.8	-	-	-	-
Sorghum Silage	-	69.6	72.2	32.5	30.5
Corn Grain	18.3	24.1	19.2	61.6	59.9
Supplement *	2.9	6.3	8.7	6.0	9.6
Fiber–Grain ratio	4.31	2.89	3.76	0.53	0.51
Dry Matter (%)	32.8	45.7	42.8	68.8	61.7
Crude Protein (%)	13.2	12.0	12.2	12.7	12.6
Acid Detergent Fiber (%)	23.9	19.0	25.3	8.4	11.3
Neutral Detergent Fiber (%)	40.3	33.9	41.5	18.1	21.8
Ash (%)	nd **	nd **	8.3	5.5	4.6

* supplement was a premix composed of Expeller Sunflower 30%CP; DDG Maize—29%CP, Mycosorb—ALLTECH; Monensin 20%—ELANCO; urea; Calcium carbonate; Rovimix Feedlot; and salt. ** not determined.

**Table 2:** Mean and standard deviation (SD) of feed efficiency and other trait values in each group.

Traits/Group	BPW		SPW1		SPW2		SF1		SF2	
*n*	69		59		70		58		70	
	Mean	SD	Mean	SD	Mean	SD	Mean	SD	Mean	SD
DMI (kgDM/day)	9.1	1.2	11.5	1.2	9.4	1.0	10.2	1.1	11.0	1.1
ADG (kg/day)	1.7	0.2	1.6	0.2	1.4	0.2	1.3	0.2	1.4	0.2
MBW (kg)	81.3	8.1	76.3	4.9	81.1	3.8	102.4	5.7	106.6	5.1
BFAT (mm)	5.4	1.5	4.8	1.5	4.7	1.2	11.8	2.5	11.2	2.3
RFI (kgDM/day)	<0.001	0.82	0.001	1.01	<0.001	0.76	0.007	0.63	<0.001	0.88
RFI range (kg/day)	(−2.32/2.04)		(−2.65/2.51)		(−2.90/1.96)		(−1.49/1.55)		(−2.28/1.32)	

DMI: dry matter intake; ADG: average daily gain; MBW: metabolic weight; BFAT: backfat thickness; RFI: residual feed intake; two cohorts of steers (SPW1; SPW2) on postweaning diets, and steers on finishing diets (SF1, SF2). The numbers refer to the years.

**Table 3:** ANOVA analysis for residual feed intake and related traits for feed efficiency groups.

	BPW			SPW			SF		
	High-RFI	Low-RFI	*p* Value	High-RFI	Low-RFI	*p* Value	High-RFI	Low-RFI	*p* Value
n	25	15		42	37		41	35	
Intake (kgMS/d)	9.75 (0.22)	8.07 (0.28)	***	11.50 (0.21)	9.13 (0.22)	***	11.56 (0.13)	9.45 (0.14)	***
ADG (kg/d)	1.74 (0.03)	1.71 (0.04)	ns	1.52(0.03)	1.55 (0.03)	ns	1.39 (0.03)	1.34 (0.03)	ns
MWt (kg)	80.41 (1.53)	84.29 (1.98)	ns	79.83 (0.73)	77.78 (0.79)	ns	105.72 (0.97)	103.34 (1.04)	ns
BFat (mm)	5.59 (0.34)	5.40 (0.44)	ns	5.07 (0.21)	4.44 (0.22)	*	10.95 (0.34)	11.28 (0.36)	ns
RFI (kg/d)	0.83 (0.09)	−1.14 (0.11)	***	1.08 (0.08)	−1.24 (0.08)	***	0.82 (0.07)	−0.95 (0.07)	***

Tukey–Kramer multiple comparison test for significance. *** *p* value < 0.001,and * *p* value < 0.05, ns: non-significative. The standard error (SE) accompanies the value in brackets.

**Table 4:** Percentage of abundances per group at phylum level (over 0.5% detected).

	Postweaning			Finishing	
Taxa (Phylum)	BPW	SPW1	SPW2	SF1	SF2
ASV (unclassified)	**50.72**	**52.58**	**47.43**	**53.97**	**62.45**
Actinomycetota (Actinobacteria)	**13.23**	**9.07**	18.36	**13.38**	**12.73**
Bacillota (Firmicutes)	**5.57**	**13.2**	**9.01**	**5.63**	**5.49**
Bacteroidota (Bacteroidetes)	**7.94**	**3.32**	**3.26**	**6.09**	2.61
Campylobacterota	0.64	2.02	1.63	0.79	0.78
Chlorobiota	0.96	1.33	1.93	-	0.49
Cyanobacteriota	0.72	1.22	1.47	0.49	**3.27**
Euglenozoa	-	-	-	2.46	1.02
Euryarchaeota	-	-	-	4.91	0.51
Fibrobacterota	0.55	-	-	-	-
Mycoplasmatota	-	-	-	0.65	-
Myxococcota	0.66	2.39	1.06	0.46	0.41
Pseudomonadota (Proteobacteria)	**15.84**	**9.24**	**11.43**	**9.35**	**8.09**
Rhodophyta	-	2.16	-	-	-
Spirochaetota	1.78	-	**2.87**	0.67	0.78
Verrucomicrobiota	-	0.63	-	0.58	-

in bold the 5 most abundant taxa within each group.

**Table 5:** Percentage of abundances per group at genus level (over 0.5% detected).

	Postweaning			Finishing	
Taxa (Genus)	BPW	SPW1	SPW2	SF1	SF2
ASV (unclassified)	51.33	54.35	48.39	56.47	63.55
*Azorhizobium*	**9.93**	**3.39**	**3.80**	3.14	**3.10**
*Mycobacterium*	**8.65**	**5.56**	**11.88**	0.51	2.11
*Prevotella*	**7.03**	**2.34**	**2.73**	**4.22**	1.52
*Chroomonas*	**2.50**	**1.87**	1.58	**3.94**	0.55
*Streptococcus*	**2.06**	**7.99**	**5.08**	1.61	**3.70**
*Borrelia*	1.39	1.48	2.51	-	-
*Myxococcus*	0.51	1.87	0.78	-	-
*Campylobacter*	0.60	1.82	0.99	0.69	0.66
*Renibacterium*	-	-	-	**6.93**	-
*Methanocorpusculum*	-	-	-	**3.47**	-
*Actinoplanes*	-	-	-	**3.26**	-
*Acetoanaerobium*	-	-	-	2.31	-
*Thermobifida*	1.06	-	**3.03**	-	**4.28**
*Cardiobacterium*	0.96	-	1.19	-	0.79
*Tetragenococcus*	0.38	-	1.15	-	-
*Streptomyces*	0.98	0.84	0.50	0.99	**2.61**
*Trichormus*	0.52	1.04	0.92	-	**2.36**
*Clostridium*	0.56	1.36	-	-	-
*Clostridioides*	-	1.08	0.93	-	-
*Pseudomonas*	0.50	0.92	0.85	-	-
*Flexibacter*	-	-	-	0.65	0.72

in bold the 5 most abundant taxa within each group.

**Table 6:** Mann–Whitney significance test * to compare diversity between high- and low-efficiency animals.

		BPW		SPW1		SPW2		SF1		SF2	
	RFI Group	Mean	*p* Value	Mean	*p* Value	Mean	*p* Value	Mean	*p* Value	Mean	*p* Value
Observed Richness	LRFI	3532	0.022	3278	0.028	8525	0.663	9116	0.336	8553	0.653
	HRFI	4649		4020		8569		9528		8978	
Simpson	LRFI	0.451	0.245	0.395	0.044	0.697	0.756	0.976	0.154	0.852	0.867
	HRFI	0.518		0.432		0.686		0.961		0.839	
Shannon	LRFI	2.615	0.213	2.358	0.044	4.549	0.903	7.649	0.296	6.145	0.788
	HRFI	3.108		2.605		4.511		7.411		6.019	

* non-parametric test, the Wilcoxon rank-sum test (Mann–Whitney). BPW: bulls in postweaning; SPW: steers in postweaning; SF: steers in finishing.

**Table 7:** Most significantly differential genera between steers on different diets (postweaning/finishing).

Over Expressed in	Phylum	Genus	Log2 FC (Year)	log2 FC (Diet)	*p*-Value (Diet)	q-Value (Diet)
Finishing	Actinomycetota	*Actinoplanes*	−7.56	−3.48	2.49 × 10^−42^	5.06 × 10^−41^
		*Nocardia*	0.92	−3.66	1.76 × 10^−59^	1.79 × 10^−57^
		*Pimelobacter*	1.97	−4.29	1.36 × 10^−44^	3.20 × 10^−43^
		*Renibacterium*	−3.31	−2.15	1.43 × 10^−10^	3.12 × 10^−10^
		*Streptomyces*	0.98	−1.77	2.63 × 10^−24^	1.43 × 10^−23^
	Bacillota (Firmicutes)	*Acetoanaerobium*	nd	−8.85	3.64 × 10^−60^	5.56 × 10^−58^
		*Acidaminococcus*	−2.11	−1.81	8.36 × 10^−25^	4.72 × 10^−24^
		*Bacillus*	−0.06	−2.11	3.53 × 10^−23^	1.68 × 10^−22^
		*Peribacillus*	0.87	−1.71	3.00 × 10^−19^	1.14 × 10^−18^
		*Psychrobacillus*	−0.18	−2.01	1.77 × 10^−12^	4.46 × 10^−12^
	Bacteroidota (Bacteroidetes)	*Aquimarina*	nd	−1.51	1.93 × 10^−18^	6.86 × 10^−18^
		*Flexibacter*	−1.15	−4.95	2.49 × 10^−43^	5.42 × 10^−42^
		*Sodaliphilus*	−4.93	−2.98	1.06 × 10^−28^	8.08 × 10^−28^
	Pseudomonadota	*Acetobacter*	0.56	−1.85	1.63 × 10^−18^	5.94 × 10^−18^
		*Fluoribacter*	1.47	−2.94	2.51 × 10^−47^	9.57 × 10^−46^
		*Gen_unclassified Wolbachieae*	−8.54	−7.28	1.92 × 10^−46^	5.87 × 10^−45^
		*Haemophilus*	−2.56	−1.64	4.57 × 10^−16^	1.35 × 10^−15^
		*Pantoea*	−0.97	−3.47	7.66 × 10^−34^	1.02 × 10^−32^
		*Pasteurella*	0.85	−2.80	1.68 × 10^−32^	1.83 × 10^−31^
		*Sphaerotilus*	−0.77	−2.15	1.82 × 10^−24^	1.01 × 10^−23^
		*Stella*	−3.71	−1.83	2.91 × 10^−18^	9.86 × 10^−18^
		*Vibrio*	0.77	−1.63	3.30 × 10^−16^	9.96 × 10^−16^
Postweaning	Actinomycetota	*Bifidobacterium*	−2.22	2.03	1.25 × 10^−23^	6.45 × 10^−23^
		*Gen_in_Corynebacteriaceae*	2.01	2.51	1.28 × 10^−23^	6.49 × 10^−23^
		*Microbispora*	2.13	2.85	1.31 × 10^−23^	6.56 × 10^−23^
		*Mycobacterium*	3.07	3.87	1.17 × 10^−30^	1.08 × 10^−29^
		*Saccharopolyspora*	0.29	1.52	1.05 × 10^−18^	3.85 × 10^−18^
		*Saccharothrix*	−1.24	1.60	8.76 × 10^−18^	2.90 × 10^−17^
		*Streptoalloteichus*	1.77	1.57	1.25 × 10^−12^	3.17 × 10^−12^
	Bacillota (Firmicutes)	*Butyrivibrio*	0.50	2.19	1.75 × 10^−52^	1.07 × 10^−50^
		*Clostridioides*	0.82	5.99	1.61 × 10^−55^	1.23 × 10^−53^
		*Enterococcus*	0.05	1.81	5.91 × 10^−28^	4.19 × 10^−27^
		*Holdemanella*	1.57	2.15	1.66 × 10^−23^	8.14 × 10^−23^
		*Hydrogenibacillus*	−0.94	1.51	3.24 × 10^−19^	1.22 × 10^−18^
		*Leuconostoc*	0.20	1.57	9.98 × 10^−21^	4.17 × 10^−20^
		*Ligilactobacillus*	1.18	3.11	2.12 × 10^−45^	5.49 × 10^−44^
		*Marinococcus*	0.66	1.90	7.76 × 10^−27^	5.04 × 10^−26^
		*Pediococcus*	−0.47	1.89	9.67 × 10^−27^	5.90 × 10^−26^
		*Thomasclavelia*	0.01	3.02	9.07 × 10^−27^	5.65 × 10^−26^
	Bacteroidota (Bacteroidetes)	*Bacteroides*	−1.64	2.86	8.50 × 10^−36^	1.44 × 10^−34^
		*Flavobacterium*	−2.72	1.55	2.75 × 10^−18^	9.54 × 10^−18^
		*Leeuwenhoekiella*	0.18	1.61	5.39 × 10^−25^	3.10 × 10^−24^
	Pseudomonadota	*Aeromonas*	1.66	1.73	3.01 × 10^−23^	1.46 × 10^−22^
		*Afipia*	nd	1.50	7.27 × 10^−20^	2.92 × 10^−19^
		*Azospirillum*	1.23	3.43	2.87 × 10^−35^	4.61 × 10^−34^
		*Bartonella*	−1.47	1.99	5.57 × 10^−27^	3.69 × 10^−26^
		*Beggiatoa*	−0.18	2.07	1.06 × 10^−23^	5.56 × 10^−23^
		*Ehrlichia*	1.49	1.54	1.10 × 10^−14^	3.03 × 10^−14^
		*Gallibacterium*	1.32	2.27	1.63 × 10^−28^	1.18 × 10^−27^
		*Gen_in_Gammaproteobacteria*	1.72	1.57	1.94 × 10^−20^	8.01 × 10^−20^
		*Gen_in_Methylococcaceae*	0.49	2.16	4.81 × 10^−33^	5.43 × 10^−32^
		*Gen_in_Rickettsiales*	1.22	2.62	2.11 × 10^−34^	3.07 × 10^−33^
		*Herbaspirillum*	0.36	2.19	1.37 × 10^−31^	1.31 × 10^−30^
		*Hyphomicrobium*	0.55	1.69	7.90 × 10^−24^	4.23 × 10^−23^
		*Hyphomonas*	0.72	2.26	3.56 × 10^−17^	1.17 × 10^−16^
		*Klebsiella*	3.28	1.59	3.21 × 10^−15^	9.05 × 10^−15^
		*Methylobacterium*	1.21	3.04	3.16 × 10^−47^	1.07 × 10^−45^
		*Methylorubrum*	nd	2.84	1.04 × 10^−22^	4.79 × 10^−22^
		*Nitrobacter*	2.72	3.17	6.09 × 10^−32^	6.20 × 10^−31^
		*Pseudoalteromonas*	nd	3.11	5.03 × 10^−30^	4.30 × 10^−29^
		*Pseudomonas*	1.70	1.98	8.95 × 10^−27^	5.65 × 10^−26^
		*Rhizobium*	1.53	2.10	3.57 × 10^−32^	3.75 × 10^−31^
		*Succinivibrio*	2.33	2.08	4.11 × 10^−20^	1.67 × 10^−19^
		*Thermochromatium*	−1.50	1.52	2.56 × 10^−14^	6.98 × 10^−14^

**Table 8:** Differential taxa between high and low feed efficiencies in bulls.

Over Expressed in	Phylum	Species	log2 FC (RFI)	*p*-Value (RFI)
High Efficiency (LRFI)	Actinomycetota	*Actinomyces israelii*	−1.62	9.12 × 10^−3^
	Bacillota	*Spe_in_Lactococcus*	−1.84	1.22 × 10^−2^
	Bacteroidota	*Marinoscillum furvescens*	−2.01	1.56 × 10^−3^
	Cyanobacteriota	*Spe_in_Anabaena*	−1.45	1.05 × 10^−3^
	Nitrospirota	*Leptospirillum ferrooxidans*	−1.08	3.27 × 10^−3^
	Phy_of_Dinophyceae	*Amphidinium carterae*	−1.19	2.20 × 10^−2^
	Phy_other sequences	*Plasmid pAC27*	−1.14	1.64 × 10^−2^
	Pseudomonadota	*Legionella jamestowniensis*	−3.47	8.18 × 10^−6^
Low Efficiency (HRFI)	Pseudomonadota	*Spe_in_Xanthomonadaceae*	1.01	2.90 × 10^−2^
		*Wolbachia* sp.	1.02	2.91 × 10^−2^
		*Legionella jordanis*	1.09	4.71 × 10^−2^
	Bacillota	*Ruminococcus bovis*	1.06	1.57 × 10^−2^
		*Spe_in_Oscillospiraceae*	1.23	4.34 × 10^−2^
	Phy_other sequences	*Plasmid RSF1010*	1.07	4.93 × 10^−3^
		*Plasmid pVA380-1*	1.30	2.42 × 10^−2^
	Phy_of_Dinophyceae	*Prorocentrum mariae-labouriae*	1.42	1.96 × 10^−2^

**Table 9:** Differential taxa between high and low feed efficiencies in steers at different diets at species levels.

Over Expressed	Diet	Phylum	Genus	log2 FC (Year)	log2 FC (RFI)	*p*-Value (Year)	*p*-Value (RFI)
High Efficiency	postweaning	Actinomycetota	*Brevibacterium*	−1.31	−1.67	5.01 × 10^−4^	3.14 × 10^−5^
			*Bifidobacterium*	−1.97	−1.06	3.28 × 10^−5^	1.36 × 10^−2^
			*Streptomyces*	−1.38	−1.06	7.19 × 10^−5^	7.16 × 10^−4^
			*Gen_in_Atopobiaceae*	NA	−1.03	ns	2.65 × 10^−3^
			*Nocardia*	−0.41	−1.01	2.74 × 10^−1^	6.32 × 10^−3^
		Bacteroidota	*Flexibacter*	−2.63	−1.26	5.88 × 10^−6^	2.00 × 10^−3^
		Euglenozoa	*Gen_in_Euglenida*	−1.40	−1.29	3.89 × 10^−3^	4.73 × 10^−3^
		Phy_other sequences	*Gen_of_Plasmid pSL1*	−0.09	−1.43	8.44 × 10^−1^	3.93 × 10^−3^
Low Efficiency		Actinomycetota	*Mycolicibacterium*	−0.94	1.57	4.70 × 10^−3^	2.93 × 10^−5^
		Pseudomonadota	*Thermochromatium*	1.58	1.23	9.83 × 10^−4^	6.77 × 10^−3^
			*Acetobacter*	1.44	1.61	3.09 × 10^−4^	5.05 × 10^−5^
High Efficiency	finishing	Actinomycetota	*Corynebacterium*	ns	−1.66	ns	6.51 × 10^−3^
			*Gen_in_Actinomycetota*	−2.29	−1.17	1.90 × 10^−5^	9.07 × 10^−3^
		Bacillota	*Hydrogenibacillus*	0.10	−1.75	ns	2.30 × 10^−4^
			*Thomasclavelia*	2.69	−1.52	1.09 × 10^−4^	1.79 × 10^−2^
			*Moorella*	ns	−1.37	ns	5.88 × 10^−4^
			*Sporolactobacillus*	ns	−1.04	ns	8.97 × 10^−3^
		Bacteroidota	*Gen_in_Bacteroidaceae*	ns	−1.63	ns	1.09 × 10^−4^
			*Flexithrix*	ns	−1.29	ns	6.21 × 10^−3^
			*Flavobacterium*	ns	−1.28	ns	3.66 × 10^−4^
			*Prevotella*	−1.28	−1.06	6.39 × 10^−3^	1.26 × 10^−2^
		Cyanobacteriota	*Phormidium*	ns	−1.71	ns	3.87 × 10^−2^
		Fibrobacterota	*Fibrobacter*	−2.09	−1.13	3.21 × 10^−4^	2.36 × 10^−2^
		Fusobacteriota	*Fusobacterium*	−0.11	−1.23	ns	6.33 × 10^−3^
		Mycoplasmatota	*Anaeroplasma*	ns	−1.39	ns	4.98 × 10^−2^
		Phy_of_Dinophyceae	*Symbiodinium*	ns	−1.03	ns	3.59 × 10^−2^
		Phy_PX clade	*Vaucheria*	ns	−1.29	ns	2.74 × 10^−3^
		Pseudomonadota	*Thermochromatium*	−2.22	−1.29	1.32 × 10^−6^	7.01 × 10^−4^
			*Afipia*	ns	−1.27	ns	2.13 × 10^−3^
			*Xanthobacter*	−1.43	−1.20	3.52 × 10^−3^	7.13 × 10^−3^
			*Acidiphilium*	ns	−1.03	ns	2.72 × 10^−3^
			*Methylomicrobium*	ns	−1.03	ns	7.15 × 10^−3^
Low Efficiency		Pseudomonadota	*Achromobacter*	−0.56	1.34	ns	2.92 × 10^−2^

## Data Availability

Restrictions apply to the availability of these data. Data were obtained by INIA and is available from the authors with INIA’s permission.

## Supplementary Materials

The following supporting information can be downloaded at https://www.mdpi.com/article/10.3390/microorganisms12122437/s1. Table S1: Mean number of sequence counts obtained by group, Table S2: Ruminal sample pH at the extraction (at 37 °C), Table S3: Percentage of ASVs taxonomically assigned at super-kingdom rank (Domain), Table S4: Percentage of ASVs taxonomy assigned (removing unassigned ASVs).
